# Factors associated with domestic violence in women: systematic ecological review

**DOI:** 10.15649/cuidarte.3857

**Published:** 2024-12-19

**Authors:** Ivone Tatiana Brito Jiménez, Nuria Rodríguez Ávila

**Affiliations:** 1 Universidad de Barcelona, Barcelona, España. ibritoji7@alumnes.ub.edu Universidad de Barcelona Universidad de Barcelona Barcelona España ibritoji7@alumnes.ub.edu; 2 Universidad de Barcelona, Barcelona, España. nrodriguez@ub.edu Universidad de Barcelona Universidad de Barcelona Barcelona España nrodriguez@ub.edu

**Keywords:** Domestic Violence, Women, Social Determinants of Health, Systematic Review, Violencia Doméstica, Mujeres, Determinantes Sociales de la Salud, Revisión Sistemática, Violência Doméstica, Mulheres, Determinantes Sociais da Saúde, Revisão Sistemática

## Abstract

**Introduction:**

Domestic violence is a multi-causal situation that impacts women, exposing them to significant structural inequalities.

**Objective:**

To identify patterns that perpetuate domestic violence in women through a comprehensive review of the literature, using the ecological model to understand the underlying factors.

**Materials and Methods:**

A systematic literature review was conducted in Spanish, English, and Portuguese on patterns associated with domestic violence against women, using the PubMed, Scopus, Sociological Abstracts, and JSTOR databases, following the PRISMA method. Relevant studies were identified and selected based on predefined criteria, and their quality was assessed.

**Results:**

Twenty-two studies were selected that met the relevance and quality criteria. The review reveals that domestic violence is perpetuated through various systems: in the microsystem, patterns such as low educational level, alcohol and drug consumption, and emotional dependence; in the mesosystem, lack of life skills, inability to make decisions, and child abuse; in the exosystem, low income, poverty, unemployment, and criminal records; and in the macrosystem, husband’s controlling behavior and society.

**Discussion:**

The comprehensive analysis from different microsystemic, mesosystemic, exosystemic, and macrosystemic perspectives reveals gaps in existing knowledge and reinforces hypotheses about the underlying mechanisms, corroborating similar problems in other studies.

**Conclusion:**

The study provides a comprehensive understanding of domestic violence by analyzing patterns from different systems. This approach guides the development of more effective and informed prevention interventions and policies.

## Resumen

**Introducción:**

La violencia doméstica es una situación multicausal que impacta a las mujeres, enfrentándolas a desigualdades estructurales significativas.

**Objetivo:**

Identificar patrones que perpetúan la violencia doméstica en mujeres mediante una revisión exhaustiva de la literatura, utilizando el modelo ecológico para comprender los factores subyacentes.

**Materiales y métodos:**

Se realizó una revisión sistemática de la literatura en español, inglés y portugués sobre patrones asociados a la violencia doméstica en mujeres, utilizando las bases de datos PubMed, Scopus, Sociological Abstracts y JSTOR, y siguiendo el método PRISMA. Se identificaron y seleccionaron estudios relevantes, aplicando criterios predefinidos y evaluando su calidad.

**Resultados:**

Se seleccionaron 22 investigaciones que cumplieron con los criterios de relevancia y calidad. La revisión revela que la violencia doméstica se perpetúa a través de diversos sistemas: en el microsistema, patrones como el bajo nivel educativo, el consumo de alcohol y drogas, y la dependencia emocional; en el mesosistema, la falta de habilidades para la vida, la incapacidad para tomar decisiones y el maltrato a los hijos; en el exosistema, bajos ingresos, pobreza, desempleo y antecedentes penales; y en el macrosistema, el comportamiento controlador del esposo y de la sociedad.

**Discusión:**

El análisis integral desde diferentes perspectivas microsistémicas, mesosistémicas, exosistémicas y macrosistémicas, revelan vacíos en el conocimiento existente y refuerza hipótesis sobre los mecanismos subyacentes, corroborando problemas similares en otros estudios.

**Conclusiones:**

El estudio proporciona una comprensión exhaustiva de la violencia doméstica al analizar patrones desde los diferentes sistemas. Este enfoque guía al desarrollo de intervenciones y políticas de prevención más efectivas y fundamentadas.

## Resumo

**Introdução:**

A violência doméstica é um situação multicausal que afecta as mulheres, confrontando-as com significativas desigualdades estruturais.

**Objetivo:**

Identificar padrões que perpetuam a violência doméstica nas mulheres através de uma revisão abrangente da literatura, utilizando o modelo ecológico para compreender os factores subjacentes.

**Materiais e métodos:**

Foi realizada uma revisão sistemática da literatura em espanhol, inglês e português sobre padrões associados à violência doméstica em mulheres, utilizando as bases de dados PubMed, Scopus, Sociological Abstracts e JSTOR, e seguindo o método PRISMA. Os estudos relevantes foram identificados e selecionados, aplicando critérios pré-definidos e avaliando sua qualidade.

**Resultados:**

Foram selecionadas 22 investigações que atenderam aos critérios de relevância e qualidade. A revisão revela que a violência doméstica se perpetua através de vários sistemas: no microssistema, padrões como baixo nível educacional, consumo de álcool e drogas e dependência emocional; no mesossistema, a falta de competências para a vida, a incapacidade de tomar decisões e os maus tratos às crianças; no exossistema, baixa renda, pobreza, desemprego e antecedentes criminais; e no macrossistema, o comportamento controlador do marido e da sociedade.

**Discussão:**

A análise abrangente sob diferentes perspectivas microssistêmica, mesossistêmica, exossistêmica e macrossistêmica revela lacunas no conhecimento existente e reforça hipóteses sobre os mecanismos subjacentes, corroborando problemas semelhantes em outros estudos.

**Conclusões:**

O estudo proporciona uma compreensão abrangente da violência doméstica através da análise de padrões de diferentes sistemas. Esta abordagem orienta o desenvolvimento de intervenções e políticas de prevenção mais eficazes e informadas.

## Introduction

Domestic violence, primarily perpetrated by men and predominantly affecting women, is a global public health concern with serious implications for the physical and mental health of millions of women^1^. Understanding the associated patterns is essential for designing effective interventions and policies that reduce its incidence and provide support to victims^2^.

Violence causes physical, psychological, and emotional harm on its victims^3^, manifesting in various aspects of their lives, in both public and private settings. It does not discriminate by political systems, socioeconomic status, religion, race, or culture, violating fundamental rights^3^,^4^ inherent to the human condition. It is estimated that approximately 18% of women between the ages of 15 and 49 have experienced physical or sexual abuse by their partner, with this figure rising to nearly 30% over their lives^5^.

The Pan American Health Organization (PAHO)^6^ defines violence against women as any form of gender-based violence that causes physical, sexual, or psychological harm, including threats, coercion, or unjustified restrictions on their freedom, whether in public or private spaces.

Bronfenbrenner's ecological model^7^ analyzes the microsystem, mesosystem, exosystem, and macrosystem levels comprehensively to understand how they contribute to the perpetuation of domestic violence. This approach examines individual and family factors in conjunction with social and cultural contexts, offering a deeper, complete view of the problem.

Heise's^8^ ecological model and Bronfenbrenner's ecological approach address the interaction of multiple levels of influence on human behavior, making both models effectively articulable for a study of domestic violence. According to Bronfenbrenner, interconnected systems, ranging from the microsystem (close relationships such as family and partners) to the macrosystem (cultural norms and values), influence behavior. Heise identifies four levels of domestic violence: individual, relational, community, and social. Therefore, domestic violence can be understood as a phenomenon influenced by social and structural factors that interact at different levels and not merely as an individual problem.

Although there are numerous studies on domestic violence, it is necessary to synthesize and systematically evaluate the available evidence. A systematic review consolidates the findings of various studies, identifies common patterns and gaps in knowledge, and provides a solid foundation for developing intervention strategies and public policies.

Therefore, the question arises: What patterns are associated with domestic violence against women, according to the ecological model, and how these patterns manifest across the different levels—microsystem, mesosystem, exosystem, and macrosystem—based on a systematic review of the existing literature?

## Material and Methods

The analysis involved an exhaustive literature review that served as an observational, retrospective, secondary research study using a qualitative approach that integrates studies addressing the same question^9^. The PRISMA methodology was used, which includes a checklist and a flow diagram to guide each stage of the process, from study identification to study inclusion in the final analysis.

A clear and specific research question was formulated, and then an exhaustive search was conducted in relevant databases using predefined criteria. The process was documented using the PRISMA flow diagram, which encompasses the identification, selection, eligibility, and inclusion of studies. An initial screening was conducted to identify relevant studies, assessing their quality through a critical review and synthesizing the findings qualitatively. Experts played a key role in guiding the formulation of the research question, defining selection criteria, recommending databases and search terms, and evaluating and interpreting the studies, ensuring the accuracy and depth of the review.

It also ensures a clear, consistent, and transparent presentation of the studies, guaranteeing a thorough selection, evaluation, and synthesis of the evidence and facilitating the reproduction and critical assessment of the results^10^. Inclusion criteria were established to include articles in English, Spanish, and Portuguese, as well as qualitative research on domestic violence published between 2013 and 2023. Articles not meeting these criteria were excluded.

A total of 725 articles on patterns associated with domestic violence were reviewed using the ecological model approach. The studies were gathered from various valuable sources, including Scopus, JSTOR, Sociological Abstracts, and PubMed, covering the period from 2013 to 2023. Search terms like “gender violence,” “violence towards women,” “domestic violence,” “violencia de género,” and “violencia doméstica” were used in combination with Boolean operators (AND, OR) to refine results. From these searches, 22 articles that met the inclusion criteria were selected using a purposive non-probabilistic selection method^11^. The data from this study are stored and accessible in the repository of the University of Magdalena^12^.

Since this study is a systematic literature review and does not involve human subjects, informed consent and ethics committee registration were not required.

## Results

**Figure1:**
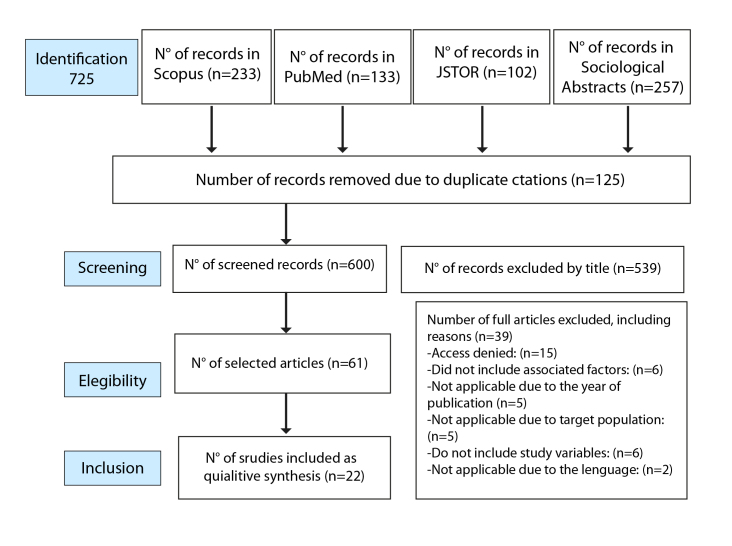
Selection diagram of the manuscripts under study.

*Source: Moher et al^13^*

As detailed in Figures 1, 725 articles were found and examined; 539 were discarded based on title, 125 were duplicates, and 61 did not meet the established inclusion criteria. After abstract review, 22 articles related to domestic violence and associated elements were selected. According to SCImago Journal Rank indicators, nine articles fall in the Q1 quartile, eleven in Q2, and eight in Q3. Patterns linked to domestic violence were identified, of which 32.10% were found in Scopus, 14.08% in JSTOR, 18.40% in PubMed, and 35.50% in Sociological Abstracts.

As evidenced in Table 1, the general matrix of the systematic literature review, the study with the largest population examined 42,002 women^14^, while the smallest involved 200 women^15^.

**Table 1:** General matrix of literature review

Author	Article	Objective	N° Women	Instruments	Results
Barbier et al.^14^	Intimate partner violence against ever-partnered women in Europe: Prevalence and associated factors—Results from the violence against women EU-wide survey.	To explore the frequency of the types of violence perpetrated by partners against women in the European Union and to seek their determinants among demographic, economic, and health-related factors.	42,002	International Survey on Violence Against Women	Women who had an immigrant father, a higher level of education, and an intellectual profession were homemakers, were drunk more than once a week, were violent in other aspects, had lower income, and were involved in relationships lasting from 1 to 10 years.
Das et al.^15^	Prevalence and associated factors of domestic violence among married women in an urban slum in South India.	To estimate the frequency of violent conditions in the domestic environment and its related factors among married women in an urban slum of Telangana.	200	Sociodemographic questionnaires, typology of violence in the domestic sphere	Older women, illiteracy, longer married life, early husband's exposure to child abuse and alcohol consumption.
Mulat et al.^16^	Assessment of domestic violence and its associated factors among ever-married reproductive-age women in Cameroon: a cross-sectional survey.	To know the prevalence of violence in the domestic environment and the linked elements among married women in Cameroon.	4903	Demographic survey applied in Cameroon in 2018	Women's educational level higher than their husbands, women exposed to media, women whose husband drinks alcohol
Kassa et al^17^	Physical violence and associated factors among women of reproductive age in Gedeo Zone, southern Ethiopia	Evaluate the typology of the conditions of physical violence and the elements linked in the reproduction stage.	588	Sociodemographic and physical violence questionnaire	Women's educational level higher than their husbands, women exposed to media, and women whose husband drinks alcohol
Karimyan et al.^18^	Comparison of associated factors of domestic violence against women by their husbands based on demographic characteristics and life skills in Iran.	Examine and compare factors linked to elements of violence in the domestic environment, considering demographic characteristics and some life skills.	640	Questionnaires aimed at violence in domestic environments	Women's education and communication skills and couples' anger management were inversely and significantly associated with domestic violence.
Aliakbari et al.^19^	Related factors of domestic violence: population-based research on Iranian women.	To evaluate the incidence of violence in domestic environments among women in Iran during 2015.	400	Questionnaires on demographic variables, obstetrics, and domestic violence	Age disparity with the husband, duration of marriage, independent income and occupation, wife's educational level, husband's addiction to psychoactive substances, marital dissatisfaction, criminal record, and experiences of violence during marriage and childhood were identified as predictive factors of violence.
Deo et al.^20^	Factors associated with domestic violence among married women residing in an urban slum.	To determine the risk factors associated with domestic violence among married women in urban slums.	385	Semi-structured questionnaire about domestic violence	Forty percent of the husbands of the women interviewed were identified as alcoholics. An association was found between low literacy, low socioeconomic status, and husband's alcohol consumption with domestic violence.
George et al.^21^	The prevalence of domestic violence and its associated factors among married women in a rural area of Puducherry, South India.	Determine the prevalence within the family environment and detect elements linked to domestic violence among married women.	310	National Family Health Survey-3	Women's illiteracy along with other factors, such as love marriage, lack of marriage registration, and duration of marriage were found to be associated with violence.
Hajian, et al.^22^	Violence against women by their intimate partners in Shahroud in northeastern region of Iran	To determine the prevalence of physical and mental violence perpetrated by men against their intimate partners and evaluate the factors related to intimate partner violence towards women in Iran.	645	WHO survey on women's health and domestic violence	Less education and a semi-manual skilled occupation of the husband, more years of marriage, excessive smoking, and use of drugs
Subhashchandr, et al.^23^	Domestic violence and its associated factors among married women in urban Chennai: A cross-sectional study.	To find the extent of domestic violence in Urban Chennai, Tamil Nadu and find the factors associated with it.	254	Sociodemographic questionnaire, types of domestic violence and factors	Women involvement in domestic decision-making, alcohol abuse among husbands, religion, and husband's education were significantly associated with domestic violence.
Pal et al.^24^	Domestic Violence against Women - An Unsolved Issue: A Community Based Study in an Urban Slum of Kolkata, India.	To estimate the frequency of domestic violence conditions among ever-married women in an urban slum neighborhood and the associated factors that contribute to it.	430	Semi-structured interview with sociodemographic characteristics	Violence was greater among women from families with low income, when the husband had a low educational level, and when wives did not adequately attend household activities.
Tesfa et al.^25^	Intimate partner violence, its sociocultural practice, and its associated factors among women in central Ethiopia.	To examine the frequency of intimate partner violence and its sociocultural context as well as related factors among married women in the Oromia region of central Ethiopia.	671	Questionnaire used in the WHO study on Women's Health and Experiences	Factors such as the husband's lack of formal education, housewife occupation, the number of children, perceived husband's dominance, having grown up in a violent domestic environment, and alcohol intake were identified as possible determinants.
Gokler et al.^26^	Prevalence of domestic violence and associated factors among married women in a semi-rural area of western Turkey.	To determine the frequency of violence in the domestic environment and related factors in married women in Türkiye.	747	Sociodemographic and marital conditions questionnaire	The youngest age group, middle/high school educational level of men, the form of first marriage, the number of children, husband's drinking and gambling habits
Lasong et al.^27^	Domestic violence among married women of reproductive age in Zimbabwe: a cross-sectional study.	To analyze the trends and elements associated with domestic violence among married women of reproductive age in Zimbabwe.	4472	The Zimbabwe Demographic and Health Surveys	Unemployed women, women who drink alcohol, women whose husbands drink alcohol, husbands who witnessed their fathers beating their mothers, husbands with more than one wife
Sapkota et al.^28^	Domestic violence and its associated factors among married women of a village development committee of rural Nepal.	Calculate the extent of the various manifestations of domestic violence and identify the elements linked to them.	355	WHO Questionnaire	Women with mental illnesses, unemployed husbands, husbands who drank alcohol, husbands who had been married more than once in their lives
Chernet et al.^29^	Prevalence of intimate partner violence against women and associated factors in Ethiopia	To determine the frequency of intimate partner violence against women and examine the linked elements in Ethiopia.	4714	Ethiopia Demographics and Health Conditions Survey 2016	Living in a rural area, getting divorced, low academic level, being between 25 and 39 years old, and living in poverty
Hussain et al.^30^	Prevalence and risk factors of domestic violence and its impacts on women’s mental health in Gilgit-Baltistan, Pakistan.	Analyze the frequency of domestic violence, the related risk factors and its effects on women's mental health.	160	Karachi Domestic Violence Conditions Scale and the Mental Health Conditions Inventory	Condition of poverty, influence of in-laws, second marriage, stepchildren, forceful sexual relations, husband’s irresponsibility, use of psychoactive substances and children with special needs
Semahegn et al.^31^	Domestic violence and its predictors among married women in reproductive age in Fagitalekoma Woreda, Awi zone, Amhara regional state, Northwestern Ethiopia.	To establish the extent of domestic violence and discern its predictors among married women of reproductive age in northwestern Ethiopia.	682	Questionnaire adapted from WHO studies on domestic violence	Husband's alcohol consumption, pregnancy, decision-making power, and annual income were predictors of domestic violence.
Colorado et al.^32^	Intimate Partner Violence and Its Associated Factors in a Sample of Colombian Immigrant Population in Spain.	Characterize gender violence among Colombian immigrants and identify its associated factors.	336	Self-reported questionnaire on conditions of gender-based violence.	Spouse's alcohol consumption, women's low educational level, younger age, and poor perception of health in Spain compared to Colombia were associated factors in men.
Gautam et al.^33^	Intimate partner violence in relation to husband characteristics and women empowerment: Evidence from Nepal.	To analyze the extent of intimate partner violence (IPV) and the linked elements among women in Nepal.	12,862	Nepal Demographic and Health Survey (NDHS) 2016	Status inconsistency, stressful life events, exposure to political violence, financial difficulties, household size, husband's controlling behavior, marital conflict, husband's marital power, poverty, social factors, acceptance of the abuse of wives.
Nuwabaine et al.^34^	Sexual violence and associated factors among women of reproductive age in Rwanda: a 2020 nationwide cross-sectional survey.	To establish the frequency of sexual violence and related elements among women of reproductive age in Rwanda.	1700	Rwanda Demographic Survey in 2020	Not participating in health decision making, having a husband/partner with primary education or no education, and having a partner who sometimes gets drunk were positively associated with sexual violence.
Tanriverdi̇ et al.^35^	Prevalence of Domestic Violence Against Married Women in Turkey and Associated Risk Factors	Analyze the prevalence of domestic violence against married women and the associated risk factors.	1105	Face-to-face interviews considering a checklist	Marital dissatisfaction, child abuse at home, lack of participation in decision making at home, residing in Kars province, and living in a large family

This section describes the factors linked to domestic violence from the ecological model conceptualized by Bronfenbrenner. The patterns found within the microsystem include the victim’s higher educational level^14^,^16^,^17^, the aggressor’s lower educational level^18^,^19^, illiteracy in both^15^,^21^,^22^, and the perpetrator's high level of education^23^. 

Perpetrator’s alcohol consumption^14^-^16^ is the most common cause, along with the use of other substances^20^,^24^,^25^, and gambling habits^9^,^24^. Furthermore, factors such as the victim being younger than the aggressor^19^,^22^,^26^, woman’ alcohol abuse^27^, and victim’s possible mental illness^28^ increase the likelihood of experiencing violence. 

Divorce is a factor associated with domestic violence^29^. However, the likelihood of experiencing it increases for people who have been married multiple times^14^,^28^,^30^, are pregnant^31^ or have several children, depending on the number of them^25^,^26^,^32^. Additionally, both a family history of violence in the victim^14^,^33^ and the aggressor’s own exposure to violence in childhood^15^,^19^,^27^ are factors associated with domestic violence. 

The dynamics that exist between domestic violence and the traditional role of housewives show a family power relationship^25^. Mass media channels also play a role in shaping attitudes that contribute to violence against women^17^, particularly when women are not involved in health decision-making^34^. Furthermore, couples married for love can experience domestic violence due to emotional and affective dependence^21^. 

As shown in Table 2, factors within the mesosystem includes a lack of life skills, taking refuge in religion, and managing emotions. The ability to communicate effectively and resolve conflicts constructively can decrease the risk of domestic violence^17^,^28^,^31^. Marital dissatisfaction is another factor; however, it does not justify or excuse violence in relationships^36^. In addition, the influence of in-laws and the children, including those with special needs^30^, can play an important role. 

Within the exosystem, the influence of socioeconomic factors such as living in areas of extreme poverty, facing economic problems influenced by social and cultural factors^15^,^16^, lack of paid work^29^,^30^, low family income^19^,^23^,^31^, unemployment^28^,^32^, having migrant parents^14^, and living in rural areas^32^ contribute to increased risk of domestic violence. Although a partner's criminal record does not guarantee or predict violence, it is associated with a heightened risk^19^. 

From the macrosystem perspective, factors include the husband's controlling behavior^24^,^25^, dominance and instilling fear in the partner^28^,^33^, as well as an extended marital life^15^. Additional risk factors are living in an environment where alcohol is consumed^32^, the perpetrator's low job status^24^, and the wife's economic, work, and financial contributions^30^. 

**Table 2:** Patterns associated with domestic violence

Author	System according to Heise's Ecological Model	Patterns associated with the victim	Patterns associated with the perpetrator
Barbier et al.^14^	Microsystem	Higher level of education	Nonexistent record
		History of violence during childhood	
	Exosystem	Alcohol and substance use	
		Cohabitation of over 10 years	
		Immigrant father	
Das et al.^15^	Microsystem	Woman's older age	Early exposure of the husband to abuse
		Illiteracy	Alcohol consumption
	Mesosystem	Long marital life	Nonexistent record
Mulat, et al.^16^	Microsystem	High educational level	Low educational level
		Husband as an alcohol consumer	Alcohol consumption
	Exosystem	Living in poverty areas	Living in poverty conditions
Kassa et al.^17^	Microsystem	Exposure to media coverage of violence against women	Low educational level
			Alcohol consumption
	Exosystem	Poverty	Poverty
Karimyan et al.^18^	Microsystem	Low educational level	Low educational level
		Lack of life skills	Nonexistent record
Aliakbari et al.^19^	Microsystem	Low educational level	Being older
			Addiction to psychoactive substances
			Experience of violence in childhood
	Exosystem	Low economic income	Low income
			Criminal record
	Mesosystem	Not registered	Marital dissatisfaction
	Macrosystem	Wife's employment	Nonexistent record
Deo et al.^20^	Microsystem	Not registered	Low literacy level
George et al.^21^	Microsystem	Illiteracy	Lack of marriage registration
		Marriage for love	Nonexistent record
Hajian, et al.^22^	Microsystem	Having primary and secondary education	Drug abuse
			Smoking
			Husbands' alcohol consumption
	Exosystem	Many years of marriage	Many years of marriage
Subhashchandra, et al.^23^	Microsystem	Young women	High educational level
			Alcohol abuse
	Mesosystem	Participation in household decision-making	Nonexistent record
	Macrosystem	Not recorded	Influence of religion
Pal et al.^24^	Microsystem	Age difference, younger women than their partners	Low educational level
	Exosystem	Belonging to a low-income family	Lack of paid work
	Macrosystem	Women who were unable to have a male child	Anger because wife left home without husband's permission
		They did not fulfill household duties.	Husband's controlling behavior
Tesfa et al.^25^	Microsystem	Number of children	Lack of higher education
		Being a homemaker	Alcohol consumption
		High educational level	
	Macrosystem	Not recorded	Husband's controlling behavior
Gokler et al.^26^	Microsystem	Woman younger than her husband	Low educational level
		Number of children	Alcohol consumption
			Gambling habits
Lasong et al.^27^	Microsystem	Women who consume alcohol	Husbands whose father used to beat their mother
			Alcohol consumption
	Macrosystem	Working women	Nonexistent record
Sapkota et al.^28^	Microsystem	Mental illnesses	Married more than once
			Alcohol consumption
	Exosystem	Low economic income	Unemployment
	Mesosystem	Not recorded	Husband's controlling behavior
Chernet et al.^29^	Microsystem	Divorce	Nonexistent record
		Having primary and secondary education	Nonexistent record
		Being younger than her husband.	Nonexistent record
	Exosystem	Living in rural areas	Living in rural areas
Hussain et al.^30^	Microsystem	Having been married more than once	Addiction to psychoactive substances
	Mesosystem	Influence of stepchildren	Nonexistent record
		Disabled children	
		Influence of in-laws	
Semahegn et al.^31^	Microsystem	Be pregnant	Alcohol consumption
	Mesosystem	Home decision-making power	Nonexistent record
	Exosystem	Not recorded	Low annual income
Colorado et al.^32^	Microsystem	Have children	
	Exosystem	Low economic income	Nonexistent record
	Macrosystem	Living in countries where alcohol is consumed.	
		Be an immigrant	
Gautam et al.^33^	Microsystem	Childhood violence	Alcohol consumption
	Macrosystem	Fear of husband	Husband's controlling behavior
Nuwabaine et al.^34^	Microsystem	Not participating in healthcare decision-making	Husband/partner with primary education or no education
			Alcohol consumption
Tanriverdi et al.^35^	Microsystem	Not having decision-making power at home	Nonexistent record
		Having many children	
	Mesosystem	Cohabitation dissatisfaction	Nonexistent record
		Child abuse at home.	

## Discussion

In the microsystem, educational level and illiteracy are closely linked to domestic violence. Studies reveal that women with low income or incomplete education are more likely to be victims of domestic violence36. This probability decreases when women earn their own income or if their partner has the same or higher educational level as them37. 

Consumption of alcohol and other toxic substances can exacerbate aggressive behavior by reducing self-control and intensifying violence38. Additionally, age disparity between victims and aggressors influences the prevalence of violence, with younger victims facing a higher likelihood of experiencing violence39. 

About 89.5% of women with depression have experienced violence40. The intergenerational transmission of violence within the home affects many women who suffer abuse in adulthood, often linked to the violence the aggressor experienced during childhood41. Furthermore, children who have been abused by their parents or family members tend to develop antisocial behaviors42. 

Domestic violence and the traditional role of women as primary family caregivers reveal underlying home power dynamics43, particularly the high proportion of women dedicated exclusively to domestic chores. In married couples, emotional dependency can intensify domestic violence, as the tendency to idealize the relationship and accept abusive behavior in the name of love contributes to abuse perpetuation44. 

In the mesosystem, factors like a lack of life skills, dependence on religion, and lack of decision-making stand out as relevant themes45. Husbands who share home decision-making responsibilities tend to exhibit less violent behavior, which is linked to women’s financial independence, many of whom are often employed46. Additionally, marital dissatisfaction can contribute to relationship violence47. Abuse of children within marriage is common, with abusers usually being children or stepchildren, parents or stepparents, and siblings or stepsiblings48. 

In the exosystem, factors such as lack of paid employment, low income, poverty, unemployment, residence in areas of extreme poverty, economic challenges, migration, and rural living conditions are identified as relevant factors for domestic violence49. Research indicates that people with criminal records for violent behavior are more likely to engage in domestic violence. In particular, men with a history of domestic violence tend to have distorted thoughts that justify the mistreatment of women50. 

In the macrosystem, the husband's controlling behavior is identified as an important factor in domestic violence51. This type of behavior can manifest itself through attitudes of domination, extreme jealousy, isolation of the partner from her support networks and control of economic resources. These power and control dynamics not only reflect cultural norms and values in some contexts, but also perpetuate gender inequalities that place women in more vulnerable situations. 

## Conclusions

The results of this systematic review, based on the ecological models of Heise and Bronfenbrenner, highlight that domestic violence arises from a complex interaction of factors at the individual, family, social, and cultural levels. Identifying patterns across the microsystemic, mesosystemic, exosystemic, and macrosystemic levels reveals the need for multifaceted interventions that address both the immediate symptoms and the long-term effects. This comprehensive approach underscores the importance of designing prevention strategies that consider the complexity of domestic violence, promote healthy relationships, address entrenched inequalities, and adapt to diverse contexts and gender approaches to improve the effectiveness of interventions.
